# MYTH: An algorithm to score intratumour heterogeneity based on alterations of DNA methylation profiles

**DOI:** 10.1002/ctm2.611

**Published:** 2021-10-14

**Authors:** Qian Liu, Lin Li, Xiaosheng Wang

**Affiliations:** ^1^ Biomedical Informatics Research Lab, School of Basic Medicine and Clinical Pharmacy China Pharmaceutical University Nanjing China; ^2^ Cancer Genomics Research Center, School of Basic Medicine and Clinical Pharmacy China Pharmaceutical University Nanjing China; ^3^ Big Data Research Institute China Pharmaceutical University Nanjing China

To the Editor:

Intratumour heterogeneity (ITH) has significant associations with tumour development and therapeutic responses. The evaluation of ITH at the methylation level may gain an advantage over that at the genetic and transcriptional levels, although such algorithms remain lacking. We proposed a novel algorithm to score methylation‐yielding tumour heterogeneity (MYTH) of a tumour sample TS, given a DNA methylation profiling dataset containing *m* genes and *t* tumour samples, as follows:

$$\begin{array}{c} \frac{1}{m-1}\sqrt{\sum_{i=1}^{m}{\left({\left(\mathrm{my}\left(G_{i},\mathrm{TS}\right)-\frac{1}{t}\sum_{j=1}^{t}\mathrm{my}(G_{i},CS_{j})\right)}^{2}-\frac{1}{m}\sum_{i=1}^{m}{\left(\mathrm{my}\left(G_{i},\mathrm{TS}\right)-\frac{1}{t}\sum_{j=1}^{t}\mathrm{my}\left(G_{i},CS_{j}\right)\right)}^{2}\right)}^{2}}\\ =\frac{1}{m-1}\sqrt{\sum_{i=1}^{m}{\left(\Delta {\left(G_{i},\mathrm{TS},CS_{j}\right)}^{2}-\frac{1}{m}\sum_{i=1}^{m}\Delta (G_{i},\mathrm{TS},CS_{j})\right)}^{2},}\\ \Delta \left(G_{i},\mathrm{TS},CS_{j}\right)=\mathrm{my}\left(G_{i},\mathrm{TS}\right)-\frac{1}{t}\sum_{j=1}^{t}\mathrm{my}(G_{i},CS_{j}), \end{array}$$

where my(*G_i_*, TS) represents the methylation level of gene *G_i_* in TS and my(*G_i_*, CS*_j_*) the methylation level of gene *G_i_* in tumour sample CS*_j_*. MYTH quantifies a tumour's ITH based on the standard deviations of the variations of gene methylation levels in the tumour from mean gene methylation levels in all tumour samples for a set of genes. The R package for the MYTH algorithm is available at the website https://github.com/XS‐Wang‐Lab/MYTH/.

To prove the effectiveness of MYTH in measuring ITH, we associated MYTH scores with clinical and phenotypic features, genomic features, antitumour immunity and drug response in 32 cancer types from The Cancer Genome Atlas (TCGA) (https://portal.gdc.cancer.gov/) (Table S1). We found that higher MYTH scores correlated with worse survival in pan‐cancer and many individual cancer types (Figure 1A). MYTH scores were significantly higher in metastatic than in primary tumours in six cancer types (Figure 1B). MYTH scores correlated positively with tumour stemness scores in pan‐cancer and 21 cancer types (Figure 1C). Moreover, MYTH scores correlated positively with proliferation signature scores in pan‐cancer and 24 cancer types (Figure 1D). MYTH scores were significantly lower in *EGFR*‐mutated than *EGFR*‐wildtype LUAD and higher in *BRAF*‐mutated than *BRAF*‐wildtype COAD (Figure 1E). In BRCA, MYTH scores were higher in basal‐like than HER2‐enriched and luminal A&B (ER+) subtypes (Figure 1E), consistent with the higher genomic ITH in basal‐like versus other subtypes of breast cancer.^1^ Altogether, these results indicate that the MYTH ITH level is an adverse prognostic factor in diverse cancers.

**FIGURE 1:**
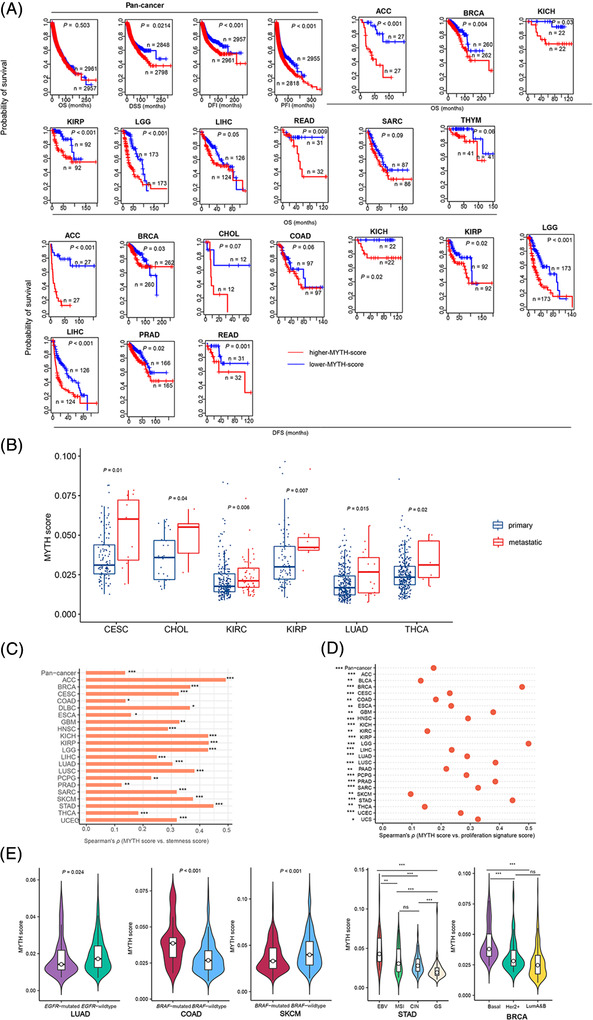
Associations of MYTH ITH with clinical features. (A) Kaplan–Meier curves showing that higher MYTH score (upper third) tumours have better survival prognosis than lower MYTH score (bottom third) tumours in pan‐cancer and multiple individual cancer types. The log‐rank test *p*‐values are shown. OS, overall survival; DSS, disease‐specific survival; PFI, progression‐free interval; DFI, disease‐free interval; DFS, disease‐free survival. (B) MYTH scores are significantly higher in metastatic than primary tumours in six cancer types. MYTH scores are significantly and positively correlated with tumour stemness scores in pan‐cancer and in 21 individual cancer types (C) and with proliferation signature scores in pan‐cancer and in 24 individual cancer types (D). The tumour stemness scores and proliferation signature scores are the ssGSEA scores of their marker genes. The Spearman correlation coefficients (*ρ*) and *p*‐values are shown in C and D. (E) Comparisons of MYTH scores between cancer subtypes. The one‐tailed Mann–Whitney *U* test *p*‐values are shown in B and E. *p < .05, **p < .01, ***p < .001; ns, not significant. ITH, intratumour heterogeneity; ACC, adrenocortical carcinoma; BLCA, bladder urothelial carcinoma; BRCA, breast invasive carcinoma; CESC, cervical squamous cell carcinoma and endocervical adenocarcinoma; CHOL, cholangiocarcinoma; COAD, colon adenocarcinoma; DLBC, lymphoid neoplasm diffuse large B‐cell lymphoma; ESCA, esophageal carcinoma; GBM, glioblastoma multiforme; HNSC, head and neck squamous cell carcinoma; KICH, kidney chromophobe; KIRC, kidney renal clear cell carcinoma; KIRP, kidney renal papillary cell carcinoma; LAML, acute myeloid leukaemia; LGG, brain lower grade glioma; LIHC, liver hepatocellular carcinoma; LUAD, lung adenocarcinoma; LUSC, lung squamous cell carcinoma; MESO, mesothelioma; PAAD, pancreatic adenocarcinoma; PCPG, pheochromocytoma and paraganglioma; PRAD, prostate adenocarcinoma; READ, rectum adenocarcinoma; SRAC, sarcoma; SKCM, skincutaneous melanoma; STAD, stomach adenocarcinoma; TGCT, testicular germ cell tumours; THCA, thyroid carcinoma; THYM, thymoma; UCEC, uterine corpus endometrial carcinoma; UCS, uterine carcinosarcoma; UVM, uveal melanoma

Tumour mutation burden (TMB) correlated positively with MYTH scores in pan‐cancer and 13 cancer types (Figure 2A). In 25 cancer types, copy number alteration scores correlated positively with MYTH scores. MYTH scores were higher in *TP53*‐mutated than *TP53*‐wildtype tumours in nine cancer types (Figure 2B). In six cancer types prevalent with MSI tumours, MYTH scores were higher in MSI‐high than MSS/MSI‐low tumours (Figure 2C). These results suggest an association between MYTH ITH and genomic instability in cancer. The enrichment scores of immune signatures (CD8+ T cells, NK cells and immune cytolytic activity) correlated inversely with MYTH scores in pan‐cancer and 24, 20 and 25 cancer types, respectively (Figure 2D), supporting that ITH inhibits antitumour immune response. MYTH scores correlated positively with tumour purity in pan‐cancer and 30 cancer types (Figure 2E), suggesting that MYTH ITH increases with the increase of tumour purity. Moreover, MYTH scores were lower in normal samples than tumour samples in pan‐cancer and eight cancer types with normal samples’ methylation data available (Figure 2F). These results reflect that MYTH does represent ITH among tumour cells.

**FIGURE 2:**
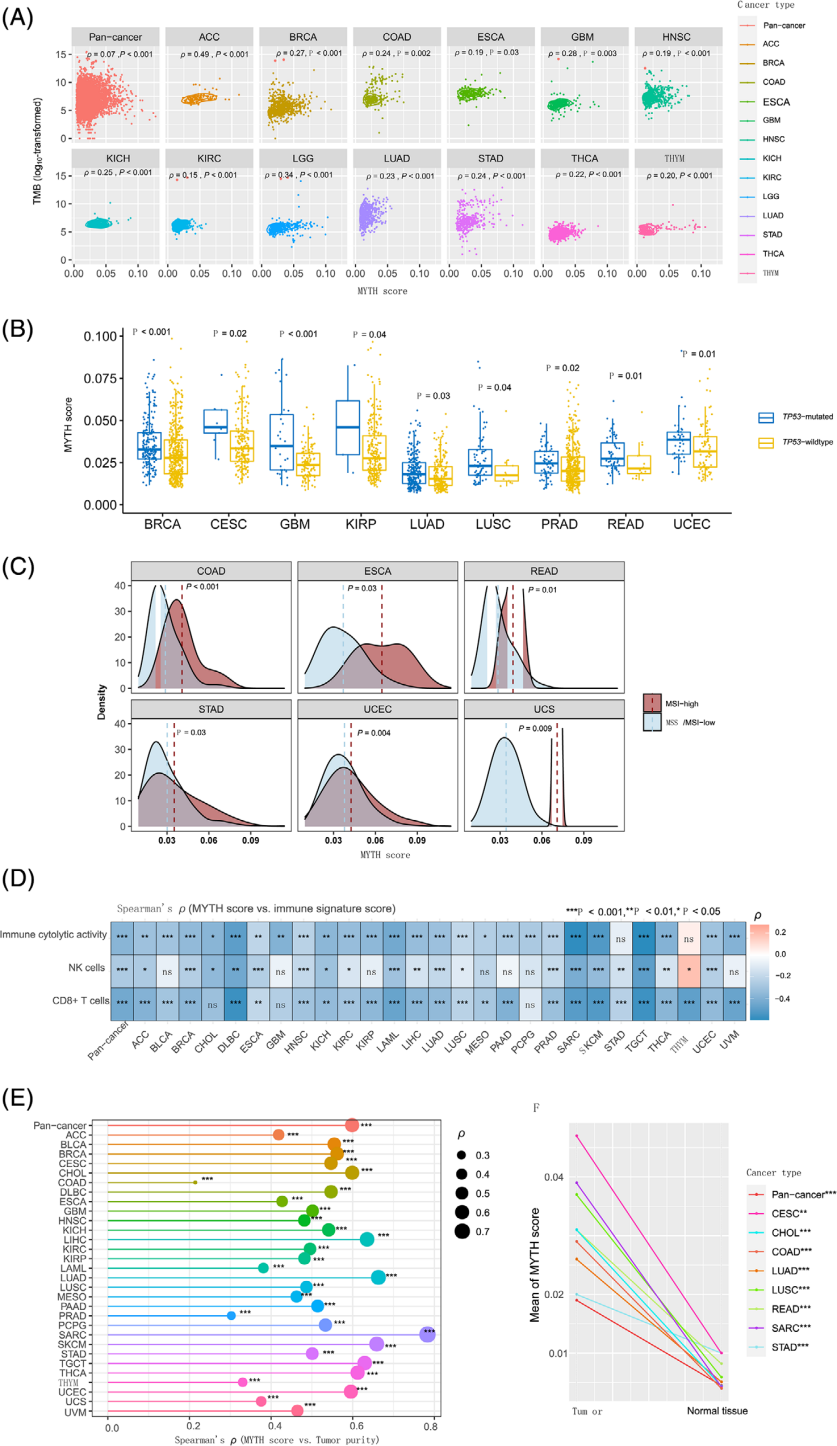
Associations of MYTH ITH with genomic instability, immune signatures and tumour purity. (A) The positive correlations between MYTH scores and TMB in pan‐cancer and in 13 individual cancer types. TMB: the total somatic mutation count in the tumour. (B) MYTH scores are significantly higher in *TP53*‐mutated than in *TP53*‐wildtype tumours in nine cancer types. (C) MYTH scores are significantly higher in MSI‐high than in MSS/MSI‐low tumours in the six cancer types harboring a high proportion of MSI tumours. The one‐tailed Mann–Whitney *U* test *p*‐values are shown in B and C. (D) The significant negative correlations between MYTH scores and the enrichment levels of three immune signatures (CD8+ T cells, NK cells and immune cytolytic activity) in pan‐cancer and in multiple individual cancer types. The enrichment levels of immune signatures are the ssGSEA scores of their marker genes. (E) The significant positive correlations between MYTH scores and tumour purity in pan‐cancer and in 30 individual cancer types. The Spearman correlation coefficients (*ρ*) and *p*‐values are shown in A, D and E. (F) MYTH scores are significantly lower in normal controls than in tumour samples in pan‐cancer and in the eight cancer types in which the DNA methylation data in normal controls are available. The one‐tailed Mann–Whitney *U* test *p*‐values are shown. *p < .05, **p < .01, ***p < .001; ns, not significant. TMB, tumour mutation burden; MSI, microsatellite instability; MSS, microsatellite stability

The global methylation levels correlated inversely with MYTH scores in 20 of the 22 cancer types with related data available^2^ (Figure 3A). WNT pathway is associated with hypermethylation in cancer,^3^ whose enrichment scores correlated inversely with MYTH scores in pan‐cancer and 22 cancer types (Figure 3B). These results suggest a negative association between MYTH scores and global methylation levels in cancer. The pathways highly enriched in high‐MYTH‐score tumours included cell cycle, p53 signalling, DNA replication and homologous recombination (Figure 3C), suggesting that increased activities of these pathways may promote ITH. The pathways highly enriched in low‐MYTH‐score tumours were mainly involved in immune and stromal signatures (Figure 3D), accordant with the negative association between MYTH scores and immune signatures.

**FIGURE 3:**
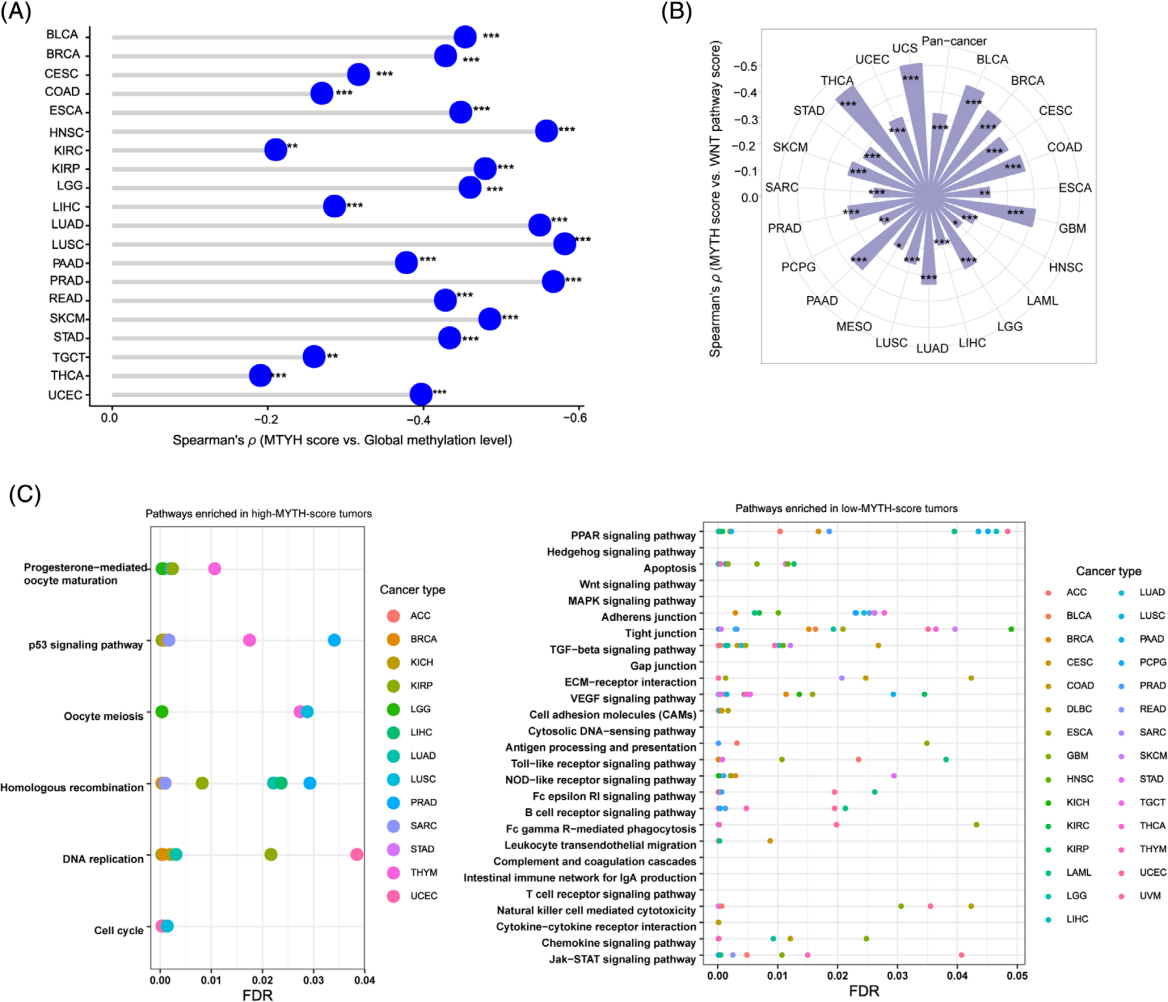
Molecular characteristics associated with MYTH ITH. (A) MYTH scores are negatively correlated with global methylation levels in 21 cancer types. The data of global methylation levels in 22 cancer types were obtained from the publication by Jung et al.^2^ (B) MYTH scores are negatively correlated with the enrichment levels of WNT pathway in pan‐cancer and in 22 individual cancer types. The enrichment levels of WNT pathway are the ssGSEA scores of all genes in the pathway. (C) Pathways highly enriched in high‐MYTH‐score (upper third) and low‐MYTH‐score (bottom third) tumours in at least eight cancer types. The Spearman correlation coefficients (*ρ*) and *p*‐values are shown in A and B. *p < .05, **p < .01, ***p < .001

We explored associations between MYTH ITH scores and ITH scores by other seven algorithms, including MATH,^4^ EXPANDS,^5^ PhyloWGS,^6^ ABSOLUTE,^7^ DEPTH,^8^ tITH^9^ and sITH.^10^ Among them, MATH, EXPANDS, PhyloWGS and ABSOLUTE evaluate ITH at the DNA level, and DEPTH, tITH and sITH at the mRNA level. In pan‐cancer, MYTH scores showed the strongest correlations with DEPTH and tITH scores and the weakest correlations with MATH and PhyloWGS scores (Figure 4A). Overall, MYTH scores had stronger correlations with ITH scores by the mRNA‐based algorithms than with those by the DNA‐based algorithms. A potential reason could be that the ITH at the DNA methylation level directly impacts mRNA expression.

**FIGURE 4:**
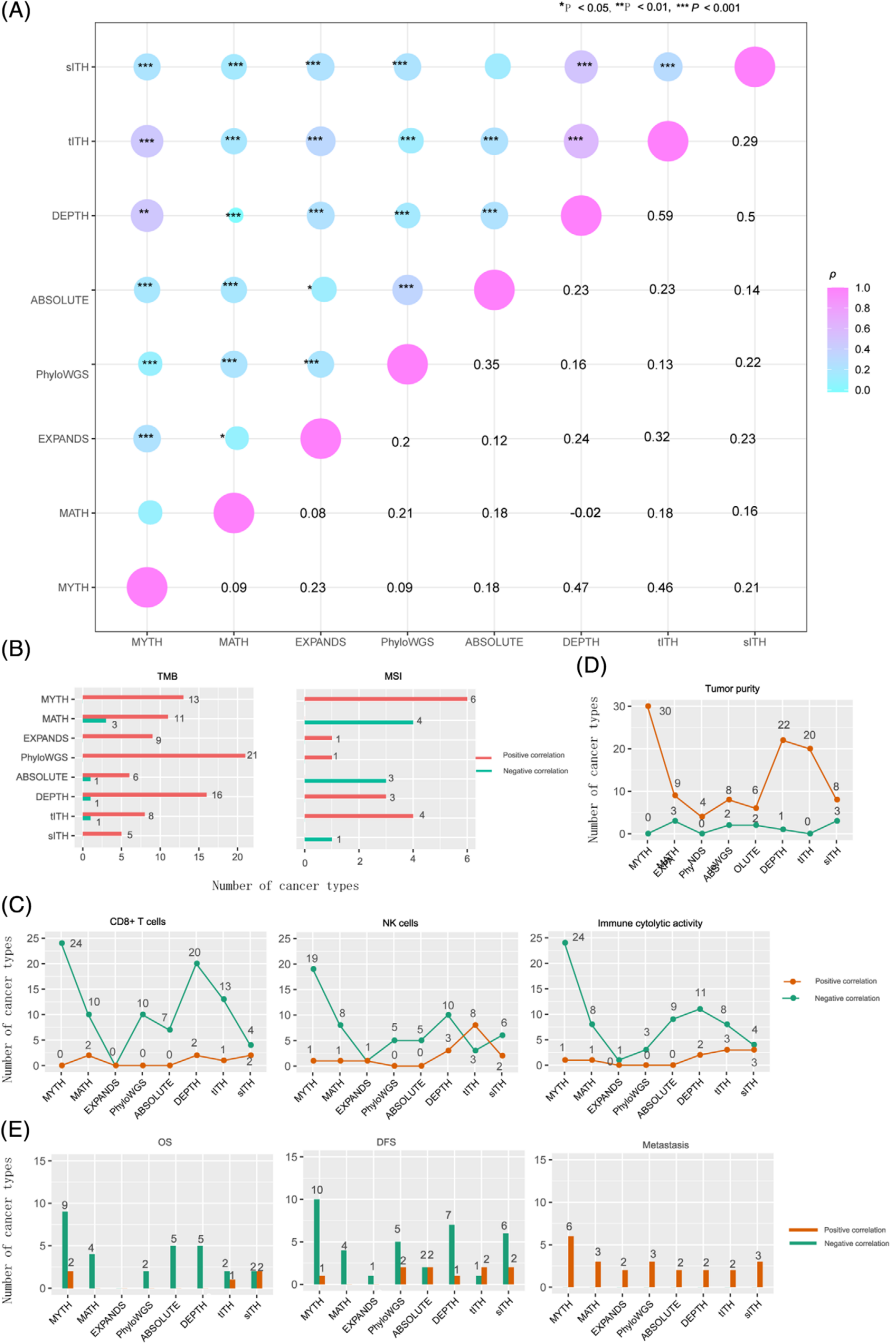
Associations and comparisons of MYTH with other seven ITH evaluation algorithms. (A) The pairwise correlations between ITH scores inferred by eight different algorithms in pan‐cancer. The Spearman correlation coefficients (*ρ*) and *p*‐values are shown. The numbers of cancer types in which ITH scores by each algorithm have significant correlations with genomic instability (B), antitumour immune signatures (C), and tumour purity (D), survival (E), and metastasis (F) among the 32 TCGA cancer types

We compared MYTH with the seven algorithms in the 32 cancer types. TMB correlated positively with ITH scores by MATH, EXPANDS, PhyloWGS, ABSOLUTE, DEPTH, tITH and sITH in 11, 9, 21, 6, 16, 8 and 5 cancer types, respectively, compared to MYTH in 13 cancer types (Figure 4B). In the six cancer types prevalent with MSI tumours, ITH scores by EXPANDS, PhyloWGS, DEPTH and tITH were significantly higher in MSI‐high than MSS/MSI‐low tumours in one, one, three and four cancer types, respectively, compared to MYTH in six cancer types (Figure 4B). In contrast, ITH scores by MYTH, ABSOLUTE and sITH were significantly lower in MSI‐high than in MSS/MSI‐low tumours in four, three and one cancer types, respectively. The immune cytolytic activity scores correlated negatively with ITH scores by MATH, EXPANDS, PhyloWGS, ABSOLUTE, DEPTH, tITH and sITH in eight, one, three, nine, eleven, eight and four cancer types, respectively, compared to MYTH in 24 cancer types (Figure 4C). Tumour purity correlated positively with ITH scores by MATH, EXPANDS, PhyloWGS, ABSOLUTE, DEPTH, tITH and sITH in nine, four, eight, six, twenty‐two, twenty and eight cancer types, respectively, compared to MYTH in 30 cancer types (Figure 4D). It indicates that MYTH is more likely to capture the ITH among tumour cells than the other algorithms. MATH scores correlated negatively with survival in more cancer types, compared to the other algorithms (Figure 4E). ITH scores by MATH, EXPANDS, PhyloWGS, ABSOLUTE, DEPTH, tITH and sITH were significantly higher in metastatic than primary tumours in three, two, three, two, two, two and three cancer types, respectively, compared to MYTH in six cancer types (Figure 4F).

In cancer cell lines, MYTH scores correlated positively with the expression of *MKI67* (a marker for cell proliferation) and DNA repair genes; MYTH scores were higher in MSI‐high than MSS/MSI‐low cell lines; MYTH scores correlated inversely with IC50 values of the compounds targeting chromatin (Figure S1). These results are consistent with the findings in the TCGA bulk tumours and suggest that higher MYTH ITH tumours are more sensitive to epigenetic therapies.

In conclusion, the MYTH algorithm is superior or comparable to established algorithms in characterising ITH.

## CONFLICT OF INTEREST

The authors declare that there is no conflict of interest.

## Supporting information


**FIGURE S1** Associations of MYTH ITH with cell proliferation, genomic instability and drug sensitivities in cancer cell lines. The significant positive correlations between MYTH scores and the expression levels of the cell proliferation marker gene *MKI67* (A) and DNA repair genes (B). (C) MYTH scores are significantly higher in MSI‐high than MSS/MSI‐low cell lines. (D) The significant negative correlations between MYTH scores and drug sensitivities (IC50 values) of the compounds targeting chromatin. The Spearman correlation coefficients (*ρ*) and *p*‐values are shown in A, B and D. The one‐tailed Mann–Whitney *U* test *p*‐values are shown in **C**. ***p < .001. The data of DNA methylation levels (gene level), gene expression profiles and MSI in cancer cell lines and drug sensitivities (IC50 values) of these cell lines to 265 compounds were from the Genomics of Drug Sensitivity in Cancer (GDSC) project (https://www.cancerrxgene.org/downloads)

**TABLE S1** A summary of the DNA methylation profiling datasets used in this study
