# Supplementary figures and images for: MYTH: An algorithm to score intratumour heterogeneity based on alterations of DNA methylation profiles
Source: Clin Transl Med. 2021 Oct 14;11(10):e611. doi: 10.1002/ctm2.611 (PMC8516364; doi:10.1002/ctm2.611)

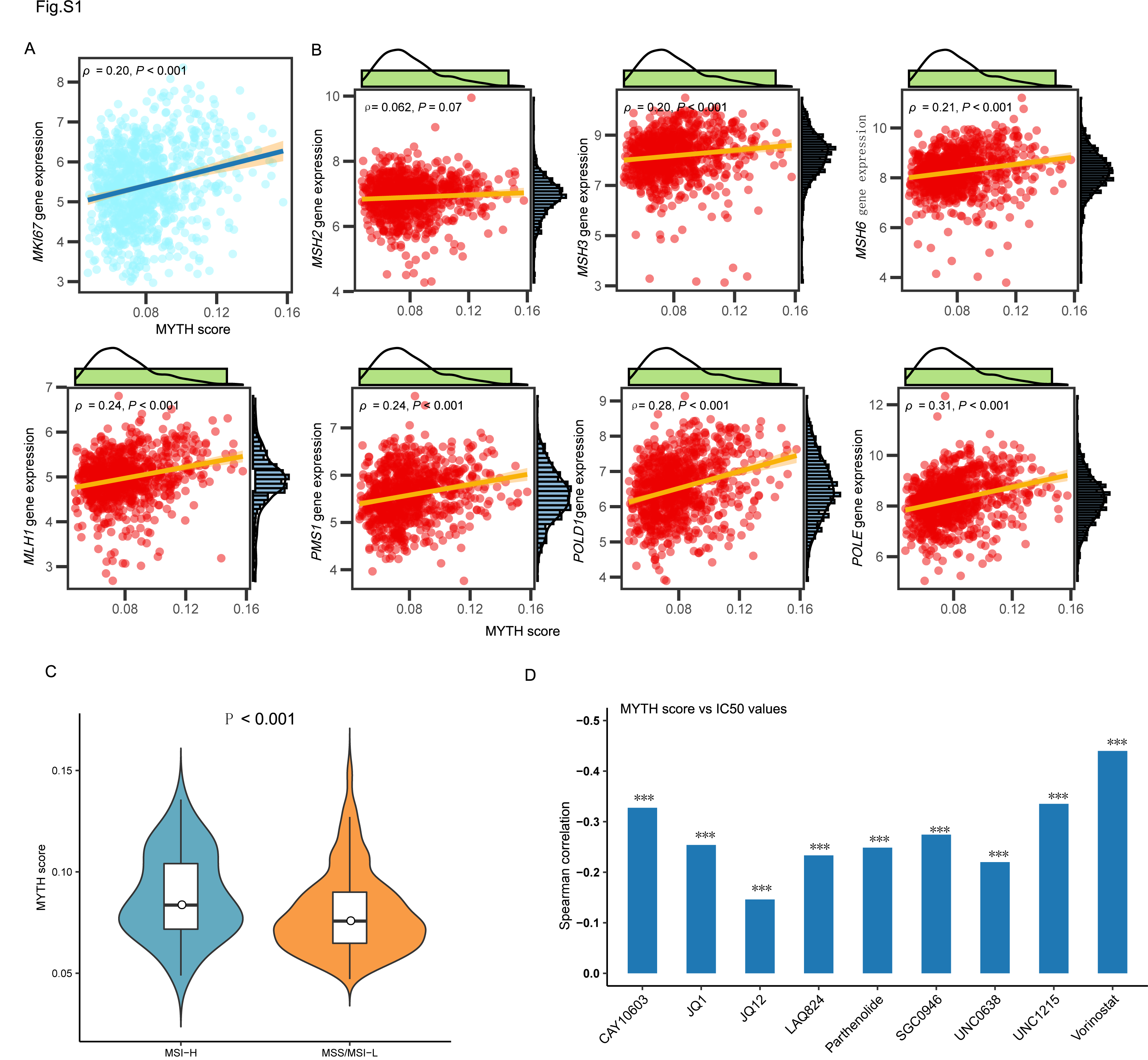

Supplement: Supplementary file 1 — FIGURE S1 Associations of MYTH ITH with cell proliferation, genomic instability and drug sensitivities in cancer cell lines. The significant positive correlations between MYTH scores and the expression levels of the cell proliferation marker gene MKI67 (A) and DNA repair genes (B). (C) MYTH scores are significantly higher in MSI‐high than MSS/MSI‐low cell lines. (D) The significant negative correlations between MYTH scores and drug sensitivities (IC50 values) of the compounds targeting chromatin. The Spearman correlation coefficients (ρ) and p‐values are shown in A, B and D. The one‐tailed Mann–Whitney U test p‐values are shown in C. ***p < .001. The data of DNA methylation levels (gene level), gene expression profiles and MSI in cancer cell lines and drug sensitivities (IC50 values) of these cell lines to 265 compounds were from the Genomics of Drug Sensitivity in Cancer (GDSC) project (https://www.cancerrxgene.org/downloads) [file CTM2-11-e611-s002.tif]
