# Supplementary material for: MYTH: An algorithm to score intratumour heterogeneity based on alterations of DNA methylation profiles
Source: Clin Transl Med. 2021 Oct 14;11(10):e611. doi: 10.1002/ctm2.611 (PMC8516364; doi:10.1002/ctm2.611)
Supplement: Supplementary file 2 — TABLE S1 A summary of the DNA methylation profiling datasets used in this study [file CTM2-11-e611-s001.docx]

**Table S1. A summary of the DNA methylation profiling datasets used in this study**

| **Cancer type** | **Cancer type (full name)** | **Platform** | **Number of all samples** | **Number of tumor samples** | **Number of normal samples** | **Sources** |
| --- | --- | --- | --- | --- | --- | --- |
| ACC | Adrenocortical Carcinoma | HM450 | 80 | 80 | 0 | TCGA (https://portal.gdc.cancer.gov/) |
| BLCA | Bladder Urothelial Carcinoma | HM450 | 413 | 413 | 0 | TCGA |
| BRCA | Breast Invasive Carcinoma | HM450 | 788 | 788 | 0 | TCGA |
| CESC | Cervical Squamous-Cell Carcinoma and Endocervical Adeno-Carcinoma | HM450 | 315 | 312 | 3 | TCGA |
| CHOL | Cholangiocarcinoma | HM450 | 45 | 36 | 9 | TCGA |
| COAD | Colon Adenocarcinoma | HM450 | 338 | 297 | 41 | TCGA |
| DLBC | Lymphoid Neoplasm Diffuse Large B-cell Lymphoma | HM450 | 48 | 48 | 0 | TCGA |
| ESCA | Esophageal Carcinoma | HM450 | 186 | 186 | 0 | TCGA |
| GBM | Glioblastoma Multiforme | HM450 | 153 | 153 | 0 | TCGA |
| HNSC | Head and Neck Squamous Cell Carcinoma | HM450 | 530 | 530 | 0 | TCGA |
| KICH | Kidney Chromophobe | HM450 | 66 | 66 | 0 | TCGA |
| KIRC | Kidney Renal Clear Cell Carcinoma | HM450 | 320 | 320 | 0 | TCGA |
| KIRP | Kidney Renal Papillary Cell Carcinoma | HM450 | 276 | 276 | 0 | TCGA |
| LAML | Acute Myeloid Leukemia | HM450 | 194 | 194 | 0 | TCGA |
| LGG | Brain Lower Grade Glioma | HM450 | 530 | 530 | 0 | TCGA |
| LIHC | Liver Hepatocellular Carcinoma | HM450 | 379 | 379 | 0 | TCGA |
| LUAD | Lung Adenocarcinoma | HM450 | 492 | 460 | 32 | TCGA |
| LUSC | Lung Squamous Cell Carcinoma | HM450 | 370 | 370 | 0 | TCGA |
| MESO | Mesothelioma | HM450 | 87 | 87 | 0 | TCGA |
| PAAD | Pancreatic Adenocarcinoma | HM450 | 185 | 185 | 0 | TCGA |
| PCPG | Pheochromocytoma and Paraganglioma | HM450 | 185 | 185 | 0 | TCGA |
| PRAD | Prostate Adenocarcinoma | HM450 | 499 | 499 | 0 | TCGA |
| READ | Rectum Adenocarcinoma | HM450 | 106 | 99 | 7 | TCGA |
| SARC | Sarcoma | HM450 | 269 | 265 | 4 | TCGA |
| SKCM | Skincutaneous Melanoma | HM450 | 473 | 473 | 0 | TCGA |
| STAD | Stomach Adenocarcinoma | HM450 | 397 | 395 | 2 | TCGA |
| TGCT | Testicular Germ-Cell Tumors | HM450 | 156 | 156 | 0 | TCGA |
| THCA | Thyroid Carcinoma | HM450 | 511 | 511 | 0 | TCGA |
| THYM | Thymoma | HM450 | 124 | 124 | 0 | TCGA |
| UCEC | Uterine Corpus Endometrial Carcinoma | HM450 | 432 | 432 | 0 | TCGA |
| UCS | Uterine Carcinosarcoma | HM450 | 57 | 57 | 0 | TCGA |
| UVM | Uveal Melanoma | HM450 | 80 | 80 | 0 | TCGA |
| Pan-cancer | Pan-cancer | HM450 | 8,906 | 8,906 | 0 | TCGA |
| Cancer cell lines | Cancer Cell Line Encyclopedia | HM450 | 1,028 | 1,028 | 0 | GDSC (https://www.cancerrxgene.org) |
